# An Approach for Security Enhancement of Certain Encryption Schemes Employing Error Correction Coding and Simulated Synchronization Errors

**DOI:** 10.3390/e24030406

**Published:** 2022-03-14

**Authors:** Miodrag J. Mihaljević, Lianhai Wang, Shujiang Xu

**Affiliations:** 1 The Shandong Provincial Key Laboratory of Computer Networks, Qilu University of Technology (Shandong Academy of Sciences), Jinan 250014, China; wanglh@sdas.org (L.W.); xushj@sdas.org (S.X.); 2 Mathematical Institute, The Serbian Academy of Sciences and Arts, 11000 Belgrade, Serbia

**Keywords:** encryption, cryptographic security enhancement, erasure error correction, channel with deletion errors, mutual information, channel capacity, the probability of classification error

## Abstract

An approach for the cryptographic security enhancement of encryption is proposed and analyzed. The enhancement is based on the employment of a coding scheme and degradation of the ciphertext. From the perspective of the legitimate parties that share a secret key, the degradation appears as a transmission of the ciphertext through a binary erasure channel. On the other hand, from the perspective of an attacker the degradation appears as a transmission of the ciphertext over a binary deletion channel. Cryptographic security enhancement is analyzed based on the capacity of the related binary deletion channel. An illustrative implemementation framework is pointed out.

## 1. Introduction

Enhancing the security of certain cryptographic primitives by employing randomness has been employed in a number of reported designs (see, e.g., [1,2]), as well as in the context of wire-tap coding. Following these approaches, two main directions have appeared. One approach is based on the employment of a cryptograhic key control of error correction encoding and decoding, given, for example, in [3,4,5,6,7]. The other approach is the employment of error-correction coding and noisy channels for cryptographic security enhancement of a given encryption scheme: This approach has been reported, for example, in [8,9,10,11,12,13,14,15].

*Motivation*. The employment of coding and noisy channel based techniques for the security enhancement of given encryption appears as an important topic. In particular, this approach could significantly increase the cryptographic security margin of a lightweight encryption scheme. On the other hand, this approach also implies additional complexity overhead. Accordingly, it appears as an interesting issue to design security enhancement with a number of parameters that provide control over desired security enhancement and required implementation and execution overheads of the encryption. The main motivation for this paper was addressing the security enhancement of a given encryption that provides the opportunity for trade-off between the security margin increasing and the required overhead.

*Summary of the Results*. This paper proposes a novel approach for the security enhancement of an encryption scheme. The proposed encryption is analyzed employing certain results of information theory. The enhancement is based on the employment of an error-correction coding scheme and degradation of the ciphertext. From the perspective of the legitimate parties that share a secret key, the degradation appears as a transmission of the ciphertext through a binary erasure channel. On the other hand, from the perspective of an attacker, the degradation appears as a transmission of the ciphertext over a binary deletion channel. The degradation is performed by employing a simulated noisy channel that consists of two sub-channels so that an additional flexibility is provided for the selection of the parameters to achieve the desired security and the enhancement overhead. Cryptographic security enhancement is analyzed based on the capacity of the related binary deletion channel. It is shown that the enhancement is a function of the following parameters: probabilities of deletion in the sub-channels, capacity of the sub-channels, and the probability of the sub-channel selection for a transmission. An illustrative implementation framework is pointed out which employs a stream cipher.

*Organization of the Paper*. A novel scheme for cryptographic security enhancement of an encryption employing error-correction coding and a simulated channel that on an attacker’s side appears as a channel with synchronization errors is proposed in Section 2. Preliminaries and background for the security evaluation are given in Section 3. Section 4 provides a cryptographic security evaluation of the proposed enhanced encryption. An illustrative approach for the implementation is discussed in Section 5. Concluding notes are given in Section 6.

## 2. Proposal for a Security Enhanced Encryption

**Figure 1:**
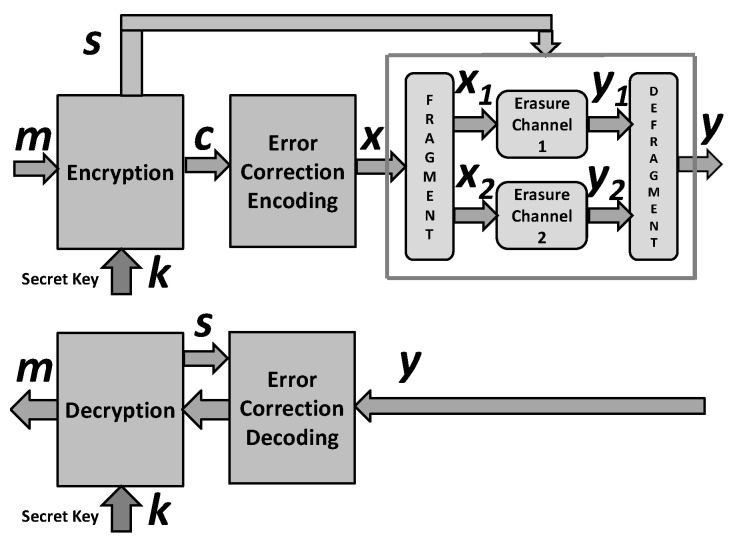
Security enhanced encryption scheme.

This section proposes the cryptographic security enhancement of an encryption scheme employing error-correction coding and a simulator of a channel with synchronization errors displayed in Figure 1.

We use the following notation. The message, a data vector subject to encryption is denoted by $m\in {\left\{0,1\right\}}^{n^{\prime }}$ and we assume that it is a realization of the binary vector variable **M**. Encrypted form of **m** is denoted by $c\in {\left\{0,1\right\}}^{n^{\prime }}$ and we assume that it is a realization of the binary vector variable C:

$$c=Enc_{k}\left(m\right)\;,$$

where $Enc_{k}\left(\cdot \right)$ denotes the encryption mapping controlled by the secret key k. The vector x denotes the encoded version of c employing an error-correction encoding Encode(·), that performs mapping ${\left\{0,1\right\}}^{n^{\prime }}\to {\left\{0,1\right\}}^{n}$, $n>n^{\prime }$:

$$x=Encode(Enc_{k}\left(m\right))\;$$

and x is a realization of a random binary variable X.

We consider a channel in which the input sequence is divided into subsequences and these subsequences are transmitted through independent i.i.d. binary deletion channels and the arrived bits after the deletion channels are combined preserving their order in the original input sequence. Consequently, the resulting channel is an i.i.d. binary deletion channel with parameters which depend on the parameters of the considered subchannels.

A simulator of the considered channel is controlled by a vector s generated by the encryption algorithm which is considered as a realization of a binary random vector S.

**Figure 2:**
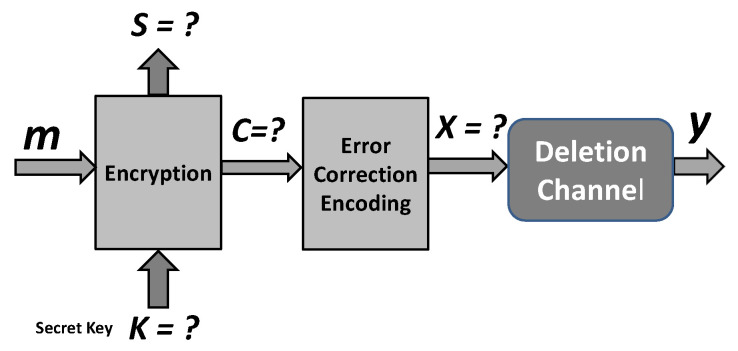
Model of encryption for cryptanalysis at the attacker’s side under known plaintext attack.

An attacker on the encryption scheme at Figure 1 faces the problem of cryptanalysis in a known plaintext attack displayed in Figure 2.

Note that the legitimate parties face the problem of decoding after a binary erasure channel, but the attacker faces a much harder problem of dealing with the decoding after a deletion channel. The knowledge of attackers is limited to the following. Each channel input bit is transmitted through Channel 1 with probability λ, and through Channel 2 with probability $\bar{\lambda }$, independently of each other. If transmitted through Channel 1 a bit is deleted with the probability $d_{1}$, and if transmitted through Channel 2 a bit is deleted with the probability *d*_2_. The attacker does not know the specific realization of the “individual channel selection events”, i.e., they do not know which specific sub-channel bit is transmitted through, and which specific sub-channel each output symbol is received from.

An illustrative instantiate of the proposed framework is given in Section 5.

## 3. Preliminaries and Background

### 3.1. Entropy, Mutual Information, and Shannon Capacity

This section provides a summary explanation on the entropy, mutual information and Shannon capacity. A random variable is denoted by an upper-case letter (e.g., *A*) and its realization is denoted by a lower-case letter (e.g., *a*). The entropy of a random object *A* is denoted by H(A), and the mutual information between two random objects *A* and *B* is denoted by I(A;B). The binary entropy function is denoted by $h\left(p\right)=-p\log_{2}p-\left(1-p\right)\log_{2}\left(1-p\right)$.

The entropy of a random variable *A* is defined as:

(1) $$H\left(A\right):=\sum_{x\in support(A)}Pr\left[A=a\right]\log_{2}\frac{1}{Pr[A=a]},$$

The mutual information I(A;B) between jointly distributed random variables *A* and *B* is defined as follows:

(2) I(A;B):=H(A)−H(A|B)=H(B)−H(B|A)

where conditional entropy is defined as:

(3) $$H\left(A|B\right)=\sum_{b\in supp(B)}Pr\left(B=b\right)H\left(A|B=b\right)$$

and:

(4) $$H\left(A|B=b\right)=\sum_{a\in supp(A)}Pr\left(A=a|B=b\right)\log_{2}\frac{1}{Pr(A=a|B=b)}$$

Consequently, the conditional mutual information when the third variable *Z* is given as:

(5) I(A,B|Z):=H(A|Z)−H(A|B,Z)=H(B|Z)−H(B|A,Z).

The Shannon capacity of a channel is denoted by *C* and is defined as:

(6) C:=sup{I(A;B)},

where *A* corresponds the channel input, *B* corresponds to the channel output, and the supremum is over the choice of the distribution of *A*.

### 3.2. Mutual Information and Capacity of the Deletion Channel with Fragmentation

**Figure 3:**
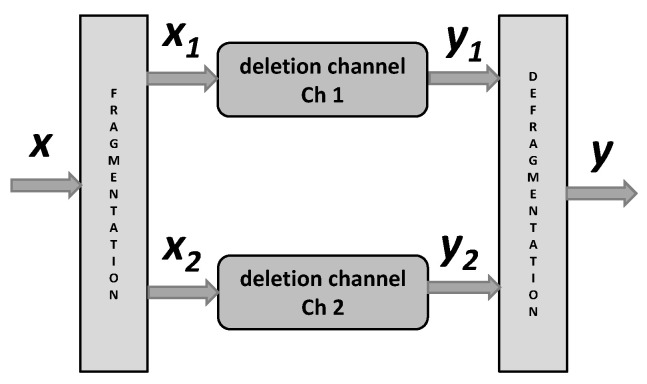
Model of the deletion channel with frangmentation.

The considered communication channel is displayed in Figure 3 and it consists of two sub-channels: $Ch_{1}$ and $Ch_{2}$.

An i.i.d. binary input deletion channel is considered in which every transmitted bit is either randomly deleted with probability *d* or received correctly with probability 1−d while there is no information about the values or the positions of the lost symbols at the transmitter or at the receiver. In the transmission of *n* symbols through the channel, the input sequence is denoted by $x=(x_{1},\ldots ,x_{n})$ in which $x_{i}\in \left\{0,1\right\}$, and $x\in {\left\{0,1\right\}}^{n}$. The output binary sequence is denoted by $y=(y_{1},\ldots ,y_{m})$ in which *m* is a realization of a binomial random variable with parameters *n* and *d* (due to the characteristics of the i.i.d. deletion channel).

Let x and y denotes input and output codewords of the considered channel, respectively.

Further on, let $x_{i}$ denotes part of the codeword x transmiied through $Ch_{i}$, i=1,2, and let $n_{i}$ denotes numbers of the codeword bits transmitted through $Ch_{i}$, i=1,2. Finally, let $y_{i}$ denotes the vector received trough $Ch_{i}$ when the channel input is $x_{i}$, i=1,2,. We assume that the vectors x, y, $x_{i}$, $y_{i}$ and $n_{i}$, are realizations of the random variables X, Y, $X_{i}$, $Y_{i}$ and $N_{i}$, respectively, i=1,2.

In continuation, we consider $I(X_{i},Y_{i})$, i=1,2, following [16]:

(7) $$\begin{array}{c} I\left(X_{i},Y_{i}\right)=I\left(X_{i},Y_{i},N_{i}\right)-I\left(X_{i},N_{i}|Y_{i}\right)\\ =I\left(X_{i},Y_{i}|N_{i}\right)+I\left(X_{i},N_{i}\right)-I\left(X_{i},N_{i}|Y_{i}\right)\\ \leq I\left(X_{i},Y_{i}|N_{i}\right)+H\left(N_{i}\right)\\ \leq I\left(X_{i},Y_{i}|N_{i}\right)+log_{2}\left(N+1\right)\\ =\sum_{n_{i}=0}^{n}P\left(N_{i}=n_{i}\right)I\left(X_{i},Y_{i}|N_{i}=n_{i}\right)+log_{2}\left(N+1\right), \end{array}$$

where in deriving the first inequality we have used the fact that:

$$H\left(N_{i}|X_{i}\right)=0\;\;\;\mathrm{and}\;\;\;I\left(X_{i},N_{i}|Y_{i}\right)\geq 0\;\;,$$

and in deriving the second equality the fact that:

(8) H(Ni)=−∑n=1NNnλnλ¯N−nlog2(Nnλnλ¯N−n)≤log2(N+1).I(Xi,Yi|Ni=ni)≤niC(di)+H(Di|Ni=ni),

where $d_{i}$ denotes the probability of deletions through the transmission of $n_{i}$ bits over the *i*-th channel and $d_{i}$, is realization of the corresponding random variable $D_{i}$, i=1,2.

Accordingly:

(9) H(Di|Ni=ni)=−∑n=1ninindind¯ini−nlog2(nindind¯ini−n)≤log2(ni+1).

and

(10) $$\begin{array}{c} I\left(X_{i},Y_{i}\right)\leq \sum_{n_{i}=0}^{n}P\left(N_{i}=n_{i}\right)\left(n_{i}C\left(d_{i}\right)+log_{2}\left(n_{i}+1\right)\right)\\ +log_{2}\left(n+1\right)\\ \leq Exp\left\{N_{i}\right\}C\left(d_{i}\right)+2log_{2}\left(n+1\right)\;, \end{array}$$

where $Exp\{N_{i}\}$ denotes the expected value of the variable $N_{i}$ and the last inequality results since $log_{2}\left(n_{i}+1\right)\leq log\left(n+1\right)$, i=1,2. Finally:

(11) $$I\left(X_{i},Y_{i}\right)\leq \lambda_{i}nC\left(d_{i}\right)+2log_{2}\left(n+1\right)\;,\;i=1,2.$$

It is shown in [16] that:

(12) $$\begin{array}{c} I\left(X,Y\right)\leq n\lambda C\left(d_{1}\right)+n\bar{\lambda }C\left(d_{2}\right)+4log_{2}\left(n+1\right)\\ +n\bar{d}log_{2}\left(\bar{d}\right)+n\lambda {\bar{d}}_{1}log_{2}\left(\lambda {\bar{d}}_{1}\right)+n\bar{\lambda }{\bar{d}}_{2}log_{2}\left(\bar{\lambda }{\bar{d}}_{2}\right)\\ =\;\;\Psi (n,\lambda ,d_{1},d_{2},C\left(d_{1}\right),C\left(d_{2}\right)) \end{array}$$

where $\bar{d}=1-d$, $d=\lambda d_{1}+\bar{\lambda }d_{2}$, $\bar{\lambda }=1-\lambda$. ${\bar{d}}_{1}=1-d_{1}$, ${\bar{d}}_{2}=1-d_{2}$.

### 3.3. The Probability of Error and the Equivocation after a Noisy Channel

Suppose the random variables *A* and *B* represent input and output messages (out of *m* possible messages), and the given conditional entropy H(A|B) represents the average amount of information lost on *A* when *B* is given. According to [17,18], for example, we have the following general upper bound on the equivocation:

(13) $$H\left(A\right)-I\left(A,B\right)\leq h\left(P_{err}\right)+P_{err}\log_{2}\left(m-1\right),$$

where h(·)≤1 is the binary entropy function and $P_{err}=1-\mathrm{Pr}\left(A=a|B=b\right)$, and following [15], when *A* is such that it has the maximum possible entropy H(A)=m, we have:

(14) $$1-\frac{I(A,B)}{m}\leq \frac{1}{m}+\frac{P_{err}}{m}\log_{2}\left(m-1\right).$$

## 4. Security Evaluation of the Enhanced Encryption

### 4.1. Security Notation

We employ a traditional approach for analyzing cryptographic security (see [19], for example) based on the following two issues: (i) a description of what a “break” of the scheme means, and (ii) a specification of the assumed power of the adversary. A cryptographic scheme is considered as a secure one in a computational sense, if for every probabilistic polynomial-time adversary A performing an attack of some specified type, and for every polynomial p(n), there exists an integer *N* such that the probability that A succeeds (where success of the attack is also well-defined) is less than $\frac{1}{p(n)}$ for every n>N. Accordingly, the following two definitions specify a security evaluation scenario and a security statement.

**Definition** **1** ([19]). *The Adversarial Indistinguishability Experiment consists of the following steps:*

*1*. *The adversary A chooses a pair of messages $\left(m_{0};m_{1}\right)$ of the same length n, and passes them on to the encryption system for encrypting.*
*2*. *A bit b∈{0,1} is chosen uniformly at random, and only one of the two messages $\left(m_{0};m_{1}\right)$, precisely $m_{b}$, is encrypted into ciphertext $\mathrm{Enc}(m_{b})$ and returned to A;*
*3*. *Upon observing $\mathrm{Enc}(m_{b})$, and without knowledge of b, the adversary A outputs a bit $b_{0}$;*
*4*. *The experiment output is defined to be 1 if $b_{0}=b$, and 0 otherwise; if the experiment output is *1, *denoted shortly as the event (A→1), we say that A has succeeded.*

**Definition** **2** ([19]). *An encryption scheme provides indistinguishable encryption in the presence of an eavesdropper, if for all probabilistic polynomial-time adversaries A:*

(15) $$\mathrm{Pr}\left[A\to 1|\mathrm{Enc}\left(m_{b}\right)\right]\leq \frac{1}{2}+\epsilon \;,$$

*where ϵ=negl(n) is a negligibly small function.*

Definitions 1 and 2 are more precisely discussed in [19].

### 4.2. Evaluation of the Security Gain

We consider the encryption/decryption scheme proposed in Section 2 which is a security enhanced scheme of a certain basic one. Our goal is to estimate the advantage of A in the indistinguishability game specified by Definition 1 when $c\leftarrow \mathrm{Enc}(m_{b})$ where c is a particular realization of C, assuming that the advantage of A is known when $m_{0}$ and $m_{1}$ are two chosen realizations of M and the corresponding realization ${c^{\prime }}_{b}$ of $C^{\prime }$ is given, i.e., the advantage of A is known for the basic (security non-enhanced) scheme.

We assume that in the corresponding statistical model, the considered encryption scheme is such that:

(16) I(S,Y)=0andI(S,Y|M)=0,

i.e., the knowledge of Y and M does not leak (provide) any information on S.

**Lemma** **1.** *We consider the advantage of the adversary A (specified by Definition 2) to win the indistinguishability game (specified by Definition 1), assuming that the mapping of m into $c^{\prime }$ is such that $\frac{1}{2}\;+\;\epsilon$ equals the advantage of the adversary to win the game. Under these assumptions:*

$$\mathrm{Pr}\left[A\to 1|Y=y\right]=\frac{1}{2}+\epsilon \cdot \delta ,\;\;\;\;$$

(17) $$\delta \;\overset{\Delta }{=}\;\mathrm{Pr}\left(X=x_{b}^{\prime \prime }|Y=y\right)\;.$$

**Proof.**  For simplicity, it is assumed that $\frac{1}{2}\;+\;\epsilon$ equals the advantage of the adversary A (specified by Definition 2) to win the indistinguishability game. Consequently, let *b* which denotes the index of the selected message by realization of the random variable *B*.

The probability Pr(B=b|Y=y) that A wins the game is determined by the following:

(18) $$\begin{array}{c} \mathrm{Pr}\left(B=b|Y=y\right)=\frac{\mathrm{Pr}(B=b,Y=y)}{\mathrm{Pr}(Y=y)}\\ =\frac{\sum_{x}\mathrm{Pr}\left(B=b,Y=y,X=x\right)}{\mathrm{Pr}(Y=y)}\\ =\frac{\sum_{x}\mathrm{Pr}\left(B=b|Y=y,X=x\right)\mathrm{Pr}\left(Y=y,X=x\right)}{\mathrm{Pr}(Y=y)}\\ =\frac{\sum_{x}\mathrm{Pr}\left(B=b|X=x\right)\mathrm{Pr}\left(Y=y,X=x\right)}{\mathrm{Pr}(Y=y)}\;. \end{array}$$

The lemma assumption implies:

(19) $$\mathrm{Pr}\left(B=b|C=c_{b}\right)=\frac{1}{2}+\epsilon \;,$$

where $c_{b}$ corresponds to the selected $m_{b}$, and:

(20) $$\mathrm{Pr}\left(B=b|X=x\right)=\frac{1}{2}\;\;\mathrm{for}\;\mathrm{any}\;c\neq c_{b}\;.$$

Note that the encoding mapping c→x is a deterministic one-to-one mapping and consequently has no impact on the advantage of adversary A, i.e., we have:

(21) $$\mathrm{Pr}\left[A\to 1|X=x\right]=\mathrm{Pr}\left[A\to 1|C=c\right]=\frac{1}{2}+\epsilon \;.$$

Consequently:

$$\mathrm{Pr}(B=b|Y=y)=\;\frac{\mathrm{Pr}\left(B=b|X=x_{b}\right)\mathrm{Pr}\left(Y=y,X=x_{b}\right)}{\mathrm{Pr}(Y=y)}\;\;+\;\frac{\sum_{x:x\neq x_{b}}\mathrm{Pr}\left(B=b|X=x\right)\mathrm{Pr}\left(Y=y,X=x\right)}{\mathrm{Pr}(Y=y)}\;,$$

Finally, we obtain:

(22) $$\begin{array}{c} \mathrm{Pr}(B=b|Y=y)=\\ \frac{\left(\frac{1}{2}+\epsilon \right)\mathrm{Pr}\left(Y=y,X=x_{b}\right)-\frac{1}{2}\mathrm{Pr}\left(Y=y,X=x_{b}\right)}{\mathrm{Pr}(Y=y)}\\ +\;\;\frac{\frac{1}{2}\sum_{x}\mathrm{Pr}\left(Y=y,X=x\right)}{\mathrm{Pr}(Y=y)}\\ =\frac{1}{2}+\epsilon \cdot \mathrm{Pr}\left(X=x_{b}|Y=y\right)\;. \end{array}$$

QED. □

Definition 1 implies that the security of an encryption scheme increases as the difference on the adversary A advantage from $\frac{1}{2}$ decreases: The factor δ<1 shows the reduction rate of the advantage, and so we call it the advantage reduction factor.

**Theorem** **1.** *We consider the adversary A (specified by Definition 2) to win the indistinguishability game (specified by Definition 1). Let the basic encryption mapping ${\left\{0,1\right\}}^{n}\to {\left\{0,1\right\}}^{n}$ of m into $c^{\prime }$, be such that $\frac{1}{2}\;+\;\epsilon$ equals the advantage of the adversary. Consequently, the advantage of the adversary A, in the security enhanced scheme specified in Section 2 is:*

(23) $$\mathrm{Pr}\left[A\to 1|Y=y\right]<\frac{1}{2}+\epsilon \cdot \frac{\Psi (n,\lambda ,d_{1},d_{2},C\left(d_{1}\right),C\left(d_{2}\right))+1}{\log_{2}\left(2^{n}-1\right)}\;\;.$$

*where:*

(24) $$\begin{array}{c} \Psi (n,\lambda ,d_{1},d_{2},C\left(d_{1}\right),C\left(d_{2}\right))=\\ \lambda C\left(d_{1}\right)+n\bar{\lambda }C\left(d_{2}\right)+4log_{2}\left(n+1\right)\\ +\;\;n\bar{d}log_{2}\left(\bar{d}\right)+n\lambda {\bar{d}}_{1}log_{2}\left(\lambda {\bar{d}}_{1}\right)+n\bar{\lambda }{\bar{d}}_{2}log_{2}\left(\bar{\lambda }{\bar{d}}_{2}\right) \end{array}$$

*and $\bar{d}=1-d$, $d=\lambda d_{1}+\bar{\lambda }d_{2}$, $\bar{\lambda }=1-\lambda$. ${\bar{d}}_{1}=1-d_{1}$, ${\bar{d}}_{2}=1-d_{2}$.*

**Proof.**  According to the (14) we have:

(25) $$1-\frac{I(X,Y)}{n}\leq \frac{1}{n}+\frac{P_{err}}{n}\log_{2}\left(2^{n}-1\right)\;,$$

and taking into account that:

(26) $$P_{err}=1-\mathrm{Pr}\left(X=x_{b}|Y=Y\right)$$

we obtain:

(27) $$\begin{array}{c} \frac{1}{n}\mathrm{Pr}\left(X=x_{b}|Y=y\right)\log_{2}\left(2^{n}-1\right)\\ \leq -1+\frac{I(X,Y)}{n}+\frac{1}{n}+\frac{1}{n}\log_{2}\left(2^{n}-1\right)\\ <\frac{I(X,Y)}{n}+\frac{1}{n}\;, \end{array}$$

and:

(28) $$\mathrm{Pr}\left(X=x_{b}|Y=y\right)<\frac{I(X,Y)+1}{\log_{2}\left(2^{n}-1\right)}\;.$$

Finally, taking into account (12) we have:

(29) $$\mathrm{Pr}\left(X=x_{b}|Y=y\right)<\frac{\Psi (n,\lambda ,d_{1},d_{2},C\left(d_{1}\right),C\left(d_{2}\right))+1}{\log_{2}\left(2^{n}-1\right)}\;.$$

Substitution of (29) into the statement of Lemma 1 yields the proof. QED. □

Lemma 1 shows that the encryption mapping m→c enhances the security because the probability that A wins the game becomes closer to $\frac{1}{2}$, which corresponds to random guessing, by the factor δ, and Theorem 1 shows that the upper bound on δ is <<1.

## 5. Notes on Implementation Issues

As an illustration, this section proposes an instantiate of the generic framework given in Section 2. This section yields particular designs for the following three main parts of the generic framework: (i) encryption scheme; (ii) coding scheme; (iii) simulated noisy channel.

**Figure 4:**
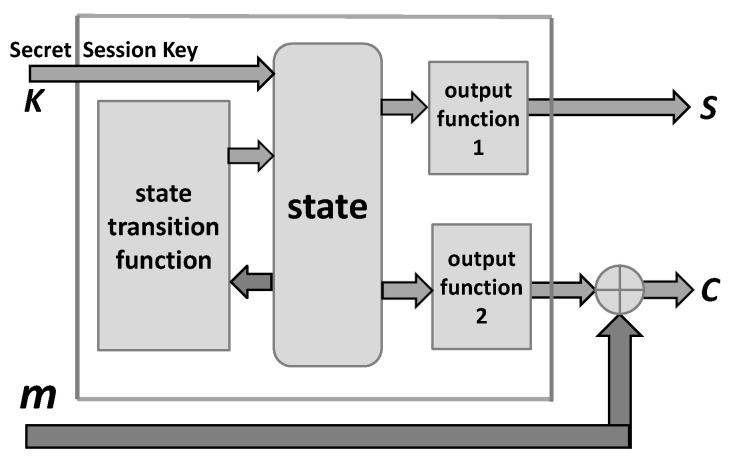
Model of encryption based on a stream cipher.

*Encryption.* The following Figure 4 displays a model of the encryption box based on a stream cipher: The inputs are the session secret key k and the plaintext message m, and the outputs are the ciphertext c and the control s of simulated noisy channel.

Note that the above scheme provides all vectors (sequences) required by encryption box in Figure 1, and in particular the vector s required for the simulation of a noisy channel.

*Coding*. As an option for suitable error correction coding we point to the LDPC codes reported in [20,21]. The time and space complexity of these codes is $O(nlog_{2}n)$ and O(n), respectively. In order to keep decoding complexity as claimed, the number of errors introduced by the simulated noisy channel should be below the error capability of the employed code, [22]. Otherwise if we are at the error-correcting capability limit we face an increase of the decoding complexity. We assume that up to Δ errors can be corrected with the claimed complexity. In a particular case as reported in [21] (Algorithm C), the time complexity will be $O(g_{max}^{2}n)$, where $g_{max}$ is a parameter, providing at the same decoding error-rate.

As an alternative option for suitable error correction coding we also point to the polar codes proposed in [23] and considered in [6,7,24], for example.

*Simulated Noisy Channel*. The simulated noisy channel box takes the sequence s as the input and performs its mapping block-by-block in order to obtain three sequences required for the simulated noisy channel composed of two binary erasure channels. Let $s^{\left(n\right)}$ denotes an *n*-bit segment of s, and let the functions $f_{i}\left(\cdot \right)$, i=1,2,3, perform mapping ${\left\{0,1\right\}}^{n}\to {\left\{0,1\right\}}^{n}$ generating the following three binary *n*-dimensional vectors:

$$\ell^{\left(n\right)}={\left[\ell_{i}\right]}_{i=1}^{n}=f_{1}\left(s^{\left(n\right)}\right),\;e^{\left(n,1\right)}={\left[e_{i}^{\left(1\right)}\right]}_{i=1}^{n}=f_{2}\left(s^{\left(n\right)}\right),\;e^{\left(n,2\right)}={\left[e_{i}^{\left(2\right)}\right]}_{i=1}^{n}=f_{3}\left(s^{\left(n\right)}\right).$$

We assume that the functions are such that the following is valid, where W(·) and Exp(·) are the vector weight and the expected value of the weight: (i) $Exp(W\left(\ell^{\left(n\right)}\right))=n\lambda$; (ii) $Exp\left(W\left(e^{\left(n,1\right)}\right)\right)=nd_{1}$; (iii) $Exp\left(W\left(e^{\left(n,2\right)}\right)\right)=nd_{2}$.

Let $x^{\left(n\right)}={\left[x_{i}\right]}_{i=1}^{n}$ be the codeword after the encoding box, and $y^{\left(n\right)}={\left[y_{i}\right]}_{i=1}^{n}$ denotes the degraded codeword after the simulated noisy channel according to the following algorithm. Please note that in order to keep the number of the erased bits within the error correction capability of the employed code, the parameter $\Delta^{*}$ is used: When the number of already erased bits is greater than $\Delta^{*}$, the probability of erasures should be reduced, and accordingly, there are two different rules regarding appearance of the output bit as “?”. Consequently, we consider the following simulator of the noisy channel.

              *Simulated Noisy Channel*

- *Input*: $x^{\left(n\right)}={\left[x_{i}\right]}_{i=1}^{n}$, the parameter $\Delta^{*}<\Delta$
- set *w* = 1.
- do *i* = 1, *n*
  - if $w\leq \Delta^{*}$
    $y_{i}$ = ? and w=w+1 if $\ell_{i}\cdot e_{i}^{\left(1\right)}=1$ or $\ell_{i}\cdot e_{i}^{\left(2\right)}=1$
    $y_{i}=x_{i}$ otherwise
  - if $w>\Delta^{*}$
    $y_{i}$ = ? if $\ell_{i}\cdot e_{i}^{\left(2\right)}=1$
    $y_{i}=x_{i}$ otherwise
- *Output*: $y^{\left(n\right)}={\left[y_{i}\right]}_{i=1}^{n}$

Note that for the legitimate receiver, $y^{\left(n\right)}$ appears as the codeword $x^{\left(n\right)}$ after the binary erasures channels. On the other hand, because the attacker does not know the sequence s, $y^{\left(n\right)}$ appears as the codeword $x^{\left(n\right)}$ after the binary deletion channels displayed in Figure 3.

## 6. Conclusions

This paper proposes a generic design for a measurable cryptographic security enhancement of certain secret key encryption schemes. This security enhancement is based on the following (see Figure 1): (i) employment of an error correction coding, (ii) splitting the codeword into two parts in the secret key dependent manner; and (iii) degradation each of the codeword parts by simulated binary erasure channels where the erasures are secret key dependent.

Note that for an attacker that does not know the secret key, the resulting channel appears as a simulated deletion channel. The security enhancement is quantified employing reported results on the capacity of the related two parallel binary deletion channels. The reported upper bound on the resulting channel capacity is established employing the upper bound on the mutual information between the inputs and outputs of the component deletion channels. The final lower bound on the achieved security gain is derived by employing relations between the probability of correct decoding and the mutual information between input and output of the resulting channel.

It is shown that the enhancement is a function of the following parameters: probabilities of deletion in the sub-channels, capacity of the sub-channels and the probability of the sub-channel selection for the transmission. Consequently, a desirable security enhancement, as well as, the implementation complexity could be achieved based on a suitable selection of the parameters related to the the employed channels and the coding scheme.

Accordingly, the main contributions of this paper are: (i) novel design of an encryption scheme which employs dedicated coding and simulated noisy channels that, from an attacker perspective, appear as binary deletion channels; and (ii) its cryptographic security evaluation, based on mutual information between input and output of certain channel with bits deletion, employing the adversarial indistinguishably experiment. It is out of the scope of this paper to discuss in detail particular implementations of the proposed framework, and so just illustrative notes are given regarding a possible implementation approach.
